# Editorial: The Grammar of Multilingualism

**DOI:** 10.3389/fpsyg.2016.01397

**Published:** 2016-09-21

**Authors:** Artemis Alexiadou, Terje Lohndal

**Affiliations:** ^1^Institute of English and American Studies, Humboldt University of BerlinBerlin, Germany; ^2^Center for General LinguisticsBerlin, Germany; ^3^Department of Language and Literature, NTNU Norwegian University of Science and TechnologyTrondheim, Norway; ^4^Department of Language and Culture, UiT The Arctic University of NorwayTromsø, Norway

**Keywords:** grammar, multilingualism, syntax, methodology, theory

Generative linguistics is primarily concerned with providing formal models of the linguistic competence of human beings. The goal is to adequately characterize and explain the structures of the grammar that each individual has constructed in his/her mind. This involves providing a formal description of the possible structures, which at the same time also rules out structures that do not occur. For example, a grammar of English should allow (1) but also rule out (2).

1. John will eat cookies tomorrow.
^*^John will cookies tomorrow eat.

The ^*^ is the indication that native speakers of English consider this sentence unacceptable. Differences between formal models need not concern us here; the important point that we want to make is that most formal models stay faithful to the following quote from Chomsky (1965, p. 3).

“Linguistic theory is concerned primarily with an ideal speaker-listener, in a completely homogeneous speech-community, who knows its language perfectly and is unaffected by such grammatically irrelevant conditions as memory limitations, distractions, shifts of attention and interest, and errors (random or characteristic) in applying his knowledge of the language in actual performance.”

Put differently, there has been an overwhelming monolingual focus within formal approaches to grammar. Although important insights have been gained from this focus, there is every reason to believe that a change of focus will prove very beneficial to formal models. More specifically, speakers who at some level of proficiency possess more than one language present a different set of data and theoretical challenges. We refer to all such speakers as “multilingual,” well aware that the group is extremely heterogeneous. For present purposes, the exact breakdown of the group is not important, but major groups include individuals who grow up with multiple native languages, second and third language learners, and heritage speakers.

One of the groundbreaking aspects of generative linguistics has been to try to answer the question of what a possible mental grammar is. Specifically, the goal has been to unearth the structures that the human mind makes use of when it comes to language and at the same time develop theories and models that exclude those structures that do not seem to occur. From this perspective, data from multilingual speakers are essential since these speakers have grammars that often interact in ways that a theory of possible mental grammars needs to incorporate.

The current research topic addresses a number of questions relating to grammatical structures in multilingual speakers as well as the methodological issues that arise in the context of studying such speakers. The majority of the papers focuses on heritage speaker bilinguals. These are speakers who are minority language speakers of a language acquired early on, which means that they are bilingual. Nevertheless, they are dominant in the majority language of the national community (see Montrul, 2008, 2016; Rothman, 2009 for much more). This leads to their characterization as unbalanced bilinguals. A typical trait of these speakers is that their grammar deviates in some way or other from the majority speakers of the relevant language. This makes it highly relevant to study which areas of the grammar are vulnerable and how this vulnerability should be understood: Is it because the acquisition of the heritage variety has been “incomplete” in some way, or is it because the grammar has attrited due to insufficient input? Some of these questions are explored in the current topic, highlighting a number of relevant factors that enter into our understanding of the nature of heritage grammars. Scontras et al'. review article focuses on the characterization of heritage speakers and what the study of these speakers can add to the study of linguistic competence. They offer a range of examples demonstrating their theoretical significance but also highlighting the methodological implications for the study of multilingualism more generally.

Corpora have become instrumental in the study of heritage speakers. Two papers contribute detailed studies of heritage speakers based on the same spoken corpus: The Corpus of American Norwegian Speech. Johannessen and Larsson study noun phrase-internal gender agreement and noun declension in a corpus of spoken American Norwegian. They argue that attrition affects agreement and not declension, and that complexity is an important factor in understanding the linguistic patterns. In the paper by Lohndal and Westergaard, gender in American Norwegian is explored further. It is shown that free-standing gender forms behave differently from suffixal declension class markers, and it is argued that transparency of gender assignment explains the vulnerability of the gender category.

Experimental methodology is pivotal in the study of multilingualism. Kim and Goodall present four formal acceptability experiments of island constructions in heritage Korean. They show that heritage speakers of Korean in the U.S. behave remarkably similar to native speakers residing in Korea, arguing that island phenomena are largely immune to environmental effects. Rather, island phenomena reveal deeper properties of the processor and/or grammar. Another experimental method is eye-tracking, which Arslan et al. use in a comparative study of how heritage speakers and late bilingual speakers of Turkish and German process grammatical evidentiality. They show that simplification takes place and they discuss how that should be interpreted theoretically.

Sometimes heritage speakers create new structures not seen in either of the two languages that are in contact. The paper by Yager et al. demonstrates exactly this point: They show that speakers of Heritage German have not simply lost dative case, rather, they have developed innovative structures to mark it, which are compatible with Universal Grammar. Again, we see the importance of studying various speaker and learner groups in order to get a better understanding of the kind of structures that the human mind is capable of generating.

Two of the papers in this research topic are concerned with language mixing in multilingual individuals. Chan considers mixing involving languages with contrasting head-complement orders, arguing that data from bilingual mixing or code-switching are highly relevant to better understand issues concerning phrase structure and linearization. Based on Persian-English bilinguals, Purmohammad conducts an experimental investigation of whether words from one of the bilingual speaker's languages can make use of the syntactic features from the other language, which he concludes is indeed possible.

Roeper is concerned with how to formally characterize the competence of multilingual speakers, notably second language speakers, arguing in favor of an approach based on Multiple Grammars. This approach holds that every speaker has a range of mental grammars, and Roeper presents numerous case-studies arguing in favor of this view. Rothman et al. are concerned with third language (L3) acquisition and how data from L3 speakers are theoretically important. They also show how L3 acquisition can benefit from employing neurolinguistics and psychological methodology to complement behavioral experiments.

Grohmann and Kambanaros are concerned with the role of language proximity, which is the closeness of the grammars that a child acquires, which they make use of to argue for an approach that they call “comparative bilingualism.” Kaltsa et al. is a detailed study of coordinate subject-verb agreement in L1 and L2 Greek, showing that bilinguals behave similarly to monolinguals in terms of sensitivity to number agreement, although bilinguals are slower in processing overall. Lastly, Garraffa et al. consider linguistic and cognitive skills in Sardinian-Italian bilingual children, demonstrating significant similarity with monolinguals, although where there are differences, they are mostly in favor of bilingual children.

## Author contributions

All authors listed, have made substantial, direct and intellectual contribution to the work, and approved it for publication.

### Conflict of interest statement

The authors declare that the research was conducted in the absence of any commercial or financial relationships that could be construed as a potential conflict of interest.
